# How to … Maximise Your Clinical Education Research Impact

**DOI:** 10.1111/tct.70382

**Published:** 2026-03-04

**Authors:** Danica Anne Sims, John Sandars, Wai Yee Amy Wong, Ray Samuriwo, Bryan Burford

**Affiliations:** ^1^ Department of Education University of Oxford Oxford UK; ^2^ Biomedical Engineering and Healthcare Technology Research Centre, Faculty of Health Sciences University of Johannesburg Johannesburg South Africa; ^3^ Medical Education, School of Medicine Edge Hill University Lancashire UK; ^4^ Medical Education, Norwich Medical School University of East Anglia Norfolk UK; ^5^ School of Health and Social Care Edinburgh Napier University Edinburgh UK; ^6^ School of Medicine Newcastle University Newcastle upon Tyne UK

## Abstract

Researchers are increasingly expected to demonstrate that their work leads to impact beyond academia, particularly when seeking research funding. Yet practical guidance on how to plan for, achieve and evidence impact in Clinical Education Research (ClinEdR) remains limited. This guide offers pragmatic advice to support postdoctoral researchers in designing fundable projects and producing work that leads to meaningful change. We argue that impact should be considered from the start of a research project, not added at the end. Early and ongoing engagement with potential research beneficiaries, including educators, learners, institutions, professional bodies, policymakers and patients, helps ensure that research addresses real‐world priorities. Such engagement can shape research questions, study design and outputs, strengthening both grant applications and the likelihood of downstream uptake. This how to guide outlines the value of articulating a clear impact goal and mapping a credible pathway to impact that distinguishes between outputs, outcomes and longer term benefits. We discuss how methodological choices can enable impact, with attention to participatory, design‐based and implementation‐focused approaches that have change embedded within them. We also highlight the importance of embedding impact checkpoints throughout a project, recognising that impact is often non‐linear, delayed and context dependent. Finally, we describe deliberate, audience‐specific dissemination strategies and practical ways to evidence impact beyond traditional academic metrics. Embedding impact thinking across the research lifecycle can enhance the relevance, reach and value of ClinEdR, while supporting postdoctoral researchers to meet funder, institutional and societal expectations.

## 1 Introduction

Researchers globally are under growing pressure from funders, universities and other key stakeholders to demonstrate that their work has ‘impact’—that it provides change or benefit *outside* academia [1]. For example, in the United Kingdom, impact is formally assessed through mechanisms such as the Research Excellence Framework (REF), where impact case studies require a clear narrative linking research to demonstrable benefit. Similar expectations exist internationally, albeit under different funding, accreditation, or accountability systems. Regardless of context, the core principles remain the same: the necessity of articulating a credible pathway to impact with evidence of how it has contributed to change or benefit. Researchers need to be able to clearly answer the ‘so what?’ question and illustrate the potential benefit of their work to others [2].

The aim of this how to guide is to support postdoctoral researchers in producing and proving that their work will have impact. In this guide, we focus primarily on planning and design‐stage decisions that maximise the likelihood of downstream impact, while highlighting how impact can be consolidated, evidenced and presented after a project concludes. We begin by defining and making a case for impact, recommending that researchers proactively embed impact throughout all phases of the research lifecycle, especially during the initial stages where engagement with potential research beneficiaries is key to illuminating what impact may look like from their varied perspectives. We then discuss the importance of effective presentation and dissemination to support impact realisation and how you can evidence the impact of your work.

## 2 What Is Impact and Why Is It Important?

Researchers, us included, can be guilty of undertaking research that *we* think is important, without much thought given to what others ‘out there’ think. We recommend an early consideration of impact, which flips this around, by putting ‘real‐world’ priorities first.

Impact therefore refers to the *effect* that research has *beyond* the academic sphere, including its direct or indirect contributions to the economy, society, culture, public policy, health and/or the environment [3]. Direct contributions are an immediate and intended effect of the research, such as changes in policy or practice, whereas indirect contributions may arise from increased awareness of and public discussion about research findings [4]. Simply put, impact can be defined as ‘beneficial change’. As societal priorities can determine the focus of publicly funded research, embedding impact into research means aligning research with publicly perceived value and meeting institutional accountability, funder expectations and local need [3].

That said, while ‘basic’ or ‘fundamental’ research remains important, in applied fields like ClinEdR, pathways to impact should always be considered. This is why *before* beginning any research project, it is essential to articulate *why the work matters* or *what change it could contribute to or facilitate*, in the short or long term. If this cannot be articulated, it is important to pause and reflect on whether the research is worth doing [5]. If impact is unclear, you may need to (re)develop your research question/s and/or approach/es—and engagement with potential research beneficiaries can assist with this.

## 3 Considering Impact Throughout All Phases of Research

**FIGURE 1:**
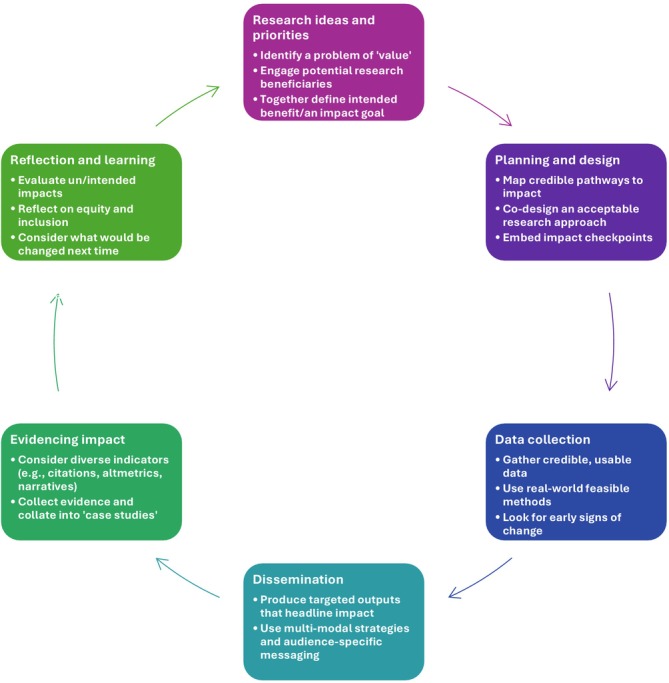
How impact can be embedded in each stage of the research lifecycle.

Impact is not something that should only be brought in at the end of a research project as an afterthought, but as a proactive consideration that influences the whole research process (Figure 1). The rest of this article will outline how this can be done throughout the research lifecycle.

## 4 Consolidating Impact During Planning and Design Phases of Research

Before beginning any research, aligning research ideas and priorities between researchers and the ‘real world’ is of central importance. It is important to consider that each potential research beneficiary (including funders) may have widely different priorities and requirements with regard to impact [6]. This is why early and ongoing engagement with these groups is essential, to determine shared priorities, varied needs and the nuance relating to specific wants, which in turn shape the formulation of research questions and onward design, explicitly aligning your research project with their expectations, values and language [7, 8, 9].

For commissioned research funding calls, where a proposal is written to meet a brief, this may be relatively straightforward. For ‘researcher led’ calls, it is important to consider what impact a funder expects to see and consult with other potential research beneficiaries as to how that may be realised. If this preparatory work is not done, there could be a risk of a misaligned project where no potential research beneficiaries are satisfied with the outcomes. In cases where stakeholders do not agree with your (or the funder's) views of ‘value’ and ‘impact’, it is imperative to be honest about these differences and find a negotiated agreement about a project's focus.

### 4.1 Develop an Impact Goal

Before jumping into data collection, it is essential to clearly define an impact or change goal (which actively includes relevant potential research beneficiary input), as this is the ‘end destination’ to which your project is moving towards [4]. An impact goal can be set by *inverting* the presenting problem to articulate the intended benefit (e.g., from ‘high diagnostic‐error rates’ to ‘improved diagnostic accuracy’) and by naming *specific* beneficiaries (e.g., students' learning may benefit from changes to clinical placements in the short term, and patient experience of care may benefit in the long term).

### 4.2 Map Credible Pathways to Maximise Impact

Defining an impact goal that takes potential research beneficiaries and time scales into account is a key step in crafting an impact pathway. A pathway to impact is a credible description of how research activities (inputs) lead to meaningful change beyond academia (via outputs and outcomes of those outputs) that is expected by specific stakeholders and beneficiaries. These pathways are often structured and visual (e.g., a table or flow chart), linking resources invested (e.g., funding) to the results (i.e., impact). The logic model is a popular choice for impact pathways because it simply and clearly sets out the progression of a research project as: activities or inputs ⟶ outputs ⟶ outcomes ⟶ impact. These roadmaps (i.e., the journey to the impact destination) explicitly show how outputs are taken up by potential research beneficiaries and translated into changes in practice or understanding.

Distinguishing between *outputs*, *outcomes* and *impact* (Table 1) helps in planning, evidencing and narrating meaningful impact [1, 3]. Significance and reach are useful for understanding how you progress from outputs to outcomes to impact: Significance speaks to the intensity of benefit, and reach the breadth or level of influence, for instance, scaling out to more people and places, or scaling up from local to national or international levels [1].

**TABLE 1:** Distinguishing between outputs, outcomes and impact.

Category	Definition	Examples
Outputs	Immediate products generated by research activities	Dataset, journal article, curriculum toolkit and so on
Outcomes	Short‐ to medium‐term changes in knowledge, attitudes, skills or practice that arise because of engagement with outputs	Educators attend faculty development and enhance their competency, toolkits are adopted in educational programmes, institutional policy is updated and so on
Impact	Longer term benefits for potential research beneficiaries, including broader society	Improved educational outcomes, enhanced patient care and national health outcomes, changes to national competency framework and healthcare policy, government budget adjustments and so on

Reviews of successful research grants show that reviewers reward realistic, short‐ to medium‐term outcomes linked to stakeholder processes (e.g., named potential research beneficiaries, iterative cocreation activities and sustained partnerships), rather than long‐term speculative ‘world‐changing’ promises [10, 11]. For this reason, the impact pathway you craft should be credible, pragmatic and clearly articulated in relation to named beneficiaries.

### 4.3 Choose Appropriate Research Methodologies

When preparing for data collection, it is essential to reflect on which methodologies have change embedded in their approaches. For example, participatory action research, design‐based research or mixed methods research with an implementation focus [12]. These methodologies move beyond discovery or description as end goals [13] and have changes in practice built into their methods.

However, projects that develop understanding can still have longer term impact enacted by others, for instance, through findings informing policy change. It is important therefore to consider *how* your research may generate knowledge which can change understanding, practice or policy. Being clear on your epistemology, and the claims that can be made from your data, is fundamental. Quantitative methods assume objective, measurable knowledge that supports generalisable statistical claims, whereas qualitative methods assume socially constructed knowledge that yields contextual, interpretive insights rather than statistical generalisation. Multiple or mixed methods can triangulate across epistemological differences [1].

The value of data is also in its validity and/or credibility. Research methods must be accessible to, and usable by, stakeholders and potential beneficiaries to ensure rigour and value. Piloting data collection with intended stakeholders in real settings can enhance the trust, acceptability, relevance and feasibility of said methods—and findings [4].

Research study design should further incorporate the potential for evaluation of impact, even if evaluation is not part of the project itself. Different study designs will provide different evaluative approaches [8, 14] and reflect epistemological choices. For example, while the impact of an educational intervention may be identified by consideration of improved examination grades or pass rates, impact on learner experience would be better examined with a qualitative design with narrative outcomes [1].

### 4.4 Embed Impact Checkpoints

If impact is to be proactively incorporated into research projects, then early, regular and real‐time data capture of impact evidence should take place throughout the research lifecycle [1, 3, 14]. Although one of the tricky aspects of impact is that it cannot be known beforehand, nor immediately after a research project has ended. Impact may be in the form of changes in knowledge, attitudes or other metrics but can also be found at a level further removed from the data, in adoption of recommendations by individuals or organisations, or application of knowledge into policy change.

Impact takes time [3] and may not be linear [5]. It can emerge through indirect, diffuse, complex, cumulative and context‐dependent pathways, shaped by a combination of the values of potential research beneficiaries, institutional dynamics and broader socio‐political factors [14]. This is why both planning for and predicting impact, in an explicit impact pathway, and intentionally searching for and recording diverse forms of impact from the start is necessary—as what counts as useful evidence may only become clear when looking back [9].

### 4.5 Disseminate Diversely and Deliberately

Once data collection and analysis are complete, effective knowledge mobilisation to societal audiences beyond immediate potential research beneficiaries, and the academic world, is an integral part of achieving impact. You should be *explicit* in communicating the benefits of your research [7, 12, 15]—do not make potential research beneficiaries guess the value of your work, tell them! Be intentional in your dissemination strategies, *tailoring messaging* to specific beneficiaries, *targeting* different audiences with communications that they find useful and compelling. Therefore, diverse, creative, multimodal and open access strategies (including Creative Commons licensed works) should be employed to maximise reach and impact to a variety of changemakers while reducing time and cognitive burden (Table 2).

**TABLE 2:** Examples of diverse, creative and multimodal dissemination strategies to a variety of potential research beneficiaries.

Potential research beneficiaries	Possible dissemination strategies	Function of dissemination
Educators	A practical toolkit providing guidance on educational approach or a faculty development workshop.	To support effective change in educational practice.
Curriculum leaders	Executive summaries with findings mapped to standards, outcomes or accreditation requirements.	To inform strategic educational agendas, decision‐making and resource allocation.
Health professionals	A summary infographic or short podcast episode might be appreciated by busy healthcare workers.	To increase awareness and acceptance of new educational approaches.
Colleagues	Amplifying the work on social media might result in the reading, using and citing of the research.	To increase awareness across disciplinary boundaries.
Patients	A short video with animations and no jargon may be an inclusive option.	To inform the wider public about changes or the reasons for changes.
Communities of practice	A free talk or collaborative event might translate and lead to the application of the research in a real‐world setting.	To introduce findings into a wider public discourse and application in real‐world settings.
The public	An accessible, plain‐language (jargon‐free) blog post or news article connecting the work to a relevant and timely issue could inform public opinion, reframe debates, set agendas and subsequently influence decision‐making and policy.	

Novel thinking about creative ways to reach different audiences may enhance impact. Think about the time and place in which different audiences may be most receptive to your findings. Increasingly, researchers are exploring arts‐based dissemination strategies though prose, poetry, visual arts, graphic novels and comics, approaches that require breaking down academic assumptions to facilitate engagement with different audiences. Conversely, for policy audiences, succinct descriptive summaries, such as policy briefings, may be the most appropriate channel. You therefore need to think about the intended audiences and avenues for dissemination from the beginning—aligning with early engagement with potential research beneficiaries [4, 7].

To disseminate deliberately, you need to be clear and intentional about the key messages that you wish to convey to minimise the risks of your research being misrepresented, misinterpreted or misused—a genuine concern in the age of artificial intelligence.

### 4.6 Demonstrate the Impact of Your Research

While we recommend the ongoing collection of impact evidence throughout the research lifecycle, we understand that post hoc evaluation often takes place. If dissemination is diverse, impact evidence would likely be diverse too, from more traditional citations through to the testimonials of potential research beneficiaries and changes in practice or policy. Critically, citations may not always be interpreted as ‘impact’ beyond academia, unless the work is being cited by the public, practitioners or policymakers (e.g., in news articles, curriculum documents and policy briefs). Therefore, ‘altmetrics’ [5, 16] should be considered as forms of impact evidence, e.g., media publicity, changed thinking and practice within the field and new stakeholder attitudes [3]. The questions in Table 3 offer guidance on where researchers might look for evidence of impact and their different forms.

**TABLE 3:** Ten questions to consider in broadening the scope of impact evidence.

Who is citing the work and where? How are the findings shaping subsequent research? How are ideas changing? Are there changes in attitudes and behaviours? Did someone repost the publication on social media? Did the journal receive a letter in response to the publication or were you invited to give a talk? Did it influence the theme of a conference or journal special edition? Was the work cited in an institutional document or a government report? Was it picked up by a news network? How many times was the podcast episode downloaded or the video viewed?

Remember to also check in with stakeholders previously engaged on whether they feel like change has been achieved and in what ways—an important aspect of all research.

## 5 Looking Back and Looking Forwards

Ongoing critical self‐reflection for evaluating the impact of past research and planning for subsequent impactful research is crucial [3]. Table 4 provides reflective prompts for research impact evaluation. If intended or expected impact is *not* achieved, why was that? Was it the study design, findings, communication or the context? What would you do differently next time?

**TABLE 4:** Eight questions for critical self‐reflection on collecting impact evidence.

Have your reasons for undertaking this work remained or evolved? Has desired change been achieved—and for who? Have stakeholders been meaningfully involved—and how? Has anyone been excluded whose inclusion would support the research design and impact? Have there been any unanticipated outcomes and impacts? Have you told a compelling story of the research, clearly communicating the benefits and value of the work? Have you reached the intended audience? Have you effectively embedded impact across the research lifecycle, and where might you improve in future work?

Lastly, designing and demonstrating impactful research should be a part of regular research team discussions and be integrated into research supervision and mentorship models—with experienced researchers guiding novice and early career researchers.

## 6 Conclusion

By embedding impact throughout the ClinEdR lifecycle—from ideation, planning and design to dissemination—the reach and benefit to learners, practitioners, systems and communities will be supported. This shift requires strategic thinking, meaningful engagement with potential research beneficiaries and a broader understanding of how knowledge is translated and used.

## Author Contributions

**Danica Anne Sims:** conceptualization, writing – original draft, writing – review and editing. **John Sandars:** conceptualization, writing – review and editing. **Wai Yee Amy Wong:** conceptualization, writing – review and editing. **Ray Samuriwo:** conceptualization, writing – review and editing. **Bryan Burford:** conceptualization, writing – review and editing.

## Funding

The authors have nothing to report.

## Ethics Statement

The authors have nothing to report.

## Consent

The authors have nothing to report.

## Conflicts of Interest

The authors declare no conflicts of interest.

## Data Availability

Data sharing is not applicable to this article as no datasets were generated or analysed during the current study.
